# Psychiatry

**Published:** 1904-05-28

**Authors:** 


### PSYCHIATRY.

*(Concluded from page 137.)*

**The Mental State in Hysteria.**—Professor Charles Dana, of New York, in an article recently published,[5] points out, after referring to the anæsthesias, paralyses, and contractures of hysteria, that the mental state with its associated crises or fit is the most profoundly established of all the syndromes of hysteria, "since in most cases it is inherent from inheritance and has simply been evoked by exciting causes." Hysteria in its graver manifestations (*H. major*) almost invariably occurs in a neuropathic subject. According to Pierre Janet,[6] with whom Dana is in agreement, the essential disorder which underlies hysteria is a cleavage or doubling (*dédoublement*) of the ego, so that the sub-conscious or automatic ego becomes dominant over the conscious or intellectual ego. The general mental state in hysteria is one of enfeeblement of energy and of volition so that there is lack of action and of initiative (aboulia) coupled with a feeling of diffidence. The obverse aspect of the mind is one of abnormal impressionability and suggestibility. There is lack of independence of character in the hysterical person, while self-consciousness and craving for sympathy are morbidly exaggerated. In persons with an hereditary tendency to insanity the symptoms of hysteria may be succeeded by morbid fears and delusions, obsessions of doubt and recurring fears (the so-called "doubting mania"), and somnambulistic attacks.

**Fragilita Osssium in the Insane.**—Dr. Maule Smith in a paper [7] read at the British Medical Association (section of Psychological Medicine) stated that an undue fragility of the bones was not infrequently met with among the insane after middle life. This change was more frequently present in females than in males, and in some forms of insanity it was more marked than in others. The ribs were by far the earliest to be affected; then followed the flat pelvic bones, the vertebræ, the skull-cap, and lastly the long bones. The morbid process was everywhere the same, viz., atrophy of the compact bony tissue and enlargement of the Haversian spaces. The change began in the bone-marrow, and spread outwards until it involved almost the entire compact tissue. An analysis of 200 cases from the post-mortem records of the West Riding Asylum was made, the strength or fragility of the ribs being estimated by testing with the hands, thus: The rib was held in a constant position between the two hands, and bent until it snapped. Microscopical examination was also made of the ribs in a certain number of cases. No fragility could be found in 26 out of 27 cases of idiocy or imbecility, or in cases of chronic mania which had proved fatal before the age of 45 years. Melancholia was marked by evidences of increased fragility of the bones, and this was still more the case in dementia, especially in female subjects. Cast into percentages the following results were obtained:

| Nature of the disease. | Percentage of undue fragility of the bones. |
|---|---|
| Dementia ... ... ... | 77·7 per cent. |
| Chronic melancholia ... | 76·4 ,, |
| Chronic mania ... ... | 66·6 ,, |
| General paralysis ... ... | 65·7 ,, |
| Epilepsy ... ... ... | 22·0 ,, |

Dr. Smith concludes from his observations on the bones that *fragilitas ossium* in the insane is rare before the age of 45 years, except in cases of general paralyis, where it is oftener met with. Fragility is looked upon as the result of a morbid change occurring in the bones of insane persons in late middle life and in old age, and this morbid change itself is probably associated with (if not due to) degenerative changes occurring in the posterior root-ganglia of the spinal cord. This was demonstrated by specimens of lantern-slides showing the pathological changes in the case of a woman, a subject of dementia, whose bones were thin and fragile and who died in the West Riding Asylum at the age of 54 years in an emaciated condition. The Nissl bodies of the posterior spinal ganglion-cells were faintly stained or, in many cases, absent, while the pericellular spaces were much infiltrated with small (connective tissue) cells. These changes were indications of serious degeneration of the ganglion-cells. The changes in the bones were secondary trophic changes following upon the degeneration of the ganglion-cells in question.

**Slighter Forms of Mental Defect in Children.**—Dr. G. E. Shuttleworth [8] in a valuable paper dealing with this subject points out that the slighter forms of mental defect or abnormality seen in children call for careful diagnosis and judicious treatment in order to prevent them from developing into graver forms of mental disability. During his three years' experience as examiner of children for admission to the special instruction schools of the London School Board, many cases of the slighter forms of mental defect were observed and studied, and "types" of cases were recognised similar to those which occurred in an exaggerated form in asylums for the insane. These cases could be classified into the following groups, viz. (*a*) sub-microcephalic cases with heads measuring in circumference not more than 19 inches (7 per cent. of the total number of cases); (*b*) cases with large heads suggestive of hydrocephaly (from 3 to 9 per cent.); (*c*) cases of Mongolian imbecility (from 2 to 3 per cent.); (*d*) scrofulous cases (over 10 per cent.); (*e*) cases with cerebral or spinal syphilis (about 2 per cent.); (*f*) cretinoid cases (from 2 to 3 per cent.); (*g*) syphilitic cases bearing characteristic stigmata (about 1 per cent.); and (*h*) a few cases of postfebrile or traumatic feeble-mindedness. There was a large group of (*k*) cases which could only be classified as "neurotic," and in many instances it was possible to trace a blending, so to say, of different types. (*l*) Epileptic cases came rather into a category by themselves for educational purposes, and of the 470 cases seen it was estimated that about 17 per cent. were fit to continue in ordinary elementary schools, whereas 27·5 per cent. were so far mentally impaired as to need separate instruction in special classes. In addition there were 40 per cent. requiring to be cared for in a resident institution for epileptics, and below these there remained a residue of 15·5 per cent., which were only fit for hospitals or asylums. The majority of the cases of mental defect were of congenital origin, though in certain types no obvious signs were noticed till some time after birth, and this was a feature with most cases of cretinism, the physical characters of which, with the exception of macroglossia, were rarely remarked before nine months of age, and in inherited syphilis it was noted also that the manifestation of symptoms was often deferred until the period of the second dentition. Rickety skulls with insufficient room for the growth and expansion of the brain were met with in pauper children and sometimes among the children of well-to-do families who reared them on patent foods and with the help of the feeding bottle. Dr. Shuttleworth described with the aid of photographs the various types of feeble-minded children alluded to above. Neurotic children were fidgety and hyperexcitable, sexual tendencies were prone to develop in them precociously, and in later childhood they were liable to suffer from "obsessions." Neurotic children were occasionally reputed to be liars when they were not consciously so, being themselves carried away by a too romantic imagination. They were sometimes thieves without apparent adequate motive, appropriating things which were of no earthly use to them, and which they freely gave away to others. Especially were such tendencies noted at the trying epoch of pubescence, though they tended to pass away after the disturbing influence of puberty had subsided. "Phobias" of various kinds were always present in neurotic children. A curious instance of this was "mysophobia," a morbid fear of soiling the hands or the dress, which caused anxious distress in the little patients, and might be compared to the hyper-conscientiousness of some pubescent girls. In the present contribution the insanities proper of childhood were not referred to, as these partook of the nature of mental disturbances such as might occur in adults, and did not connote a congenital mental defect.

[5] Medical Record, Sept. 20, 1902. [6] The Mental State of the Hysterical, 1900. [7] The Lancet, Aug. 22, 1903, p. 538. [8] Ibid., p. 538-39.

